# Prevalence and quality of life associated with erectile dysfunction and lower urinary tract symptoms: a cross-sectional study

**DOI:** 10.1590/1516-3180.2024.0045.R1.03072024

**Published:** 2024-12-20

**Authors:** Felipe Jun Kijima, Liz Cristina Motta, Jean Sousa Cavalcante, Lucas Jordão, Carlos Eduardo Dias Olioze, Plinio Takashi Karubi Palavicini Santos, João Carlos Leite da Cruz, Luis Expedito Sabage, Gabriel Araújo Medeiros, Alessandra Mazzo, Aguinaldo Cesar Nardi

**Affiliations:** I Faculdade de Medicina de Bauru (FMBRU), Universidade de São Paulo (USP), Bauru (SP), Brazil.; II Faculdade de Medicina de Bauru (FMBRU), Universidade de São Paulo (USP), Bauru (SP), Brazil.; III Faculdade de Medicina de Bauru (FMBRU), Universidade de São Paulo (USP), Bauru (SP), Brazil.; IV Faculdade de Medicina de Bauru (FMBRU), Universidade de São Paulo (USP), Bauru (SP), Brazil.; V Faculdade de Medicina de Bauru (FMBRU), Universidade de São Paulo (USP), Bauru (SP), Brazil.; VI Faculdade de Medicina de Bauru (FMBRU), Universidade de São Paulo (USP), Bauru (SP), Brazil.; VII Faculdade de Medicina de Bauru (FMBRU), Universidade de São Paulo (USP), Bauru (SP), Brazil.; VIII Faculdade de Medicina de Bauru (FMBRU), Universidade de São Paulo (USP), Bauru (SP), Brazil.; IX Faculdade de Medicina de Bauru (FMBRU), Universidade de São Paulo (USP), Bauru (SP), Brazil.; X Professor, Faculdade de Medicina de Bauru (FMBRU), Universidade de São Paulo (USP), Bauru (SP), Brazil.; XI Professor, Faculdade de Medicina de Bauru (FMBRU), Universidade de São Paulo (USP), Bauru (SP), Brazil.

**Keywords:** Lower urinary tract symptoms., Erectile dysfunction., Quality of life., Prevalence., Men’s health., Genitourinary health., Questionnaires., Health promotion.

## Abstract

**BACKGROUND:**

Genitourinary health significantly affects the quality of life of men, particularly those in middle age. Recent studies have shown that more than half of the men aged over 40 years experience some degree of low urinary tract symptoms (LUTS) or erectile dysfunction (ED).

**OBJECTIVE:**

To assess the prevalence of ED and LUTS in middle-aged men and correlate this with quality of life data.

**DESIGN AND SETTING:**

A cross-sectional study was conducted in a municipality in the countryside of São Paulo.

**METHODS:**

A trained team collected data between July 2021 and August 2022 through face-to-face interviews using a characterization instrument, International Prostate Symptom Score (IPSS), International Index of Erectile Function-6 (IIEF-6), and World Health Organization Quality-of-Life Scale.

**RESULTS:**

The study included 375 male participants with a median age of 53 years (interquartile range [IQR] 38.5-66). The IIEF-6 showed the presence of some degree of ED in 51.1% (n = 188) of patients, with a median score of 25 (IQR 21-29). The IPSS revealed that 35.2% (n = 132) of the patients had some degree of LUTS, with a median score of 5 (IQR 2-11). The urological questionnaires had a direct proportional correlation with age (P < 0.001) and significant differences between the medians of different marital statuses (P < 0.001). The presence or severity of these disorders was inversely correlated with the individuals’ quality of life (P < 0.001).

**CONCLUSIONS:**

ED and LUTS significantly correlated with the quality of life, marital status, and age in men.

## INTRODUCTION

The registration rates for men in primary healthcare services are low within the Brazilian National Health System (SUS), suggesting that fewer men utilize these services and, as a result, participate in fewer preventive and health-promoting initiatives.^1^ Consequently, the disparity in healthcare engagement contributes to the life expectancy of men, which is 7.1 years shorter than that of women. This also correlates with a higher incidence of illness among men and elevated financial burden on the healthcare system, thereby constituting a significant public health concern.^2,3^

Lower urinary tract symptoms (LUTS) and erectile dysfunction (ED) have been recognized and treated since ancient times, with records of early interventions found in Egyptian and Greco-Roman texts.^4,5^ These conditions are critically important for men’s health because of their high prevalence and significant impact on their quality of life. The Brazil LUTS study reported that 75% of men over 40 years of age experienced some degree of LUTS.^6^ Similarly, an American study found that the combined prevalence of minimum, moderate, and complete erectile dysfunction was 52%.^7^

The pathophysiology of these conditions often involves overlapping mechanisms of endothelial and nerve dysfunctions and impaired blood flow, causing symptoms that affect daily life chronically, such as pollakiuria, nocturia, urinary urgency, voiding (obstructive) symptoms (straining, weak stream, intermittent stream, and incomplete emptying), or post-micturition symptoms (post-micturition dribbling).^8^ Despite their prevalence and impact, social taboos and a cultural tendency among men to perceive themselves as invulnerable contribute to a reluctance to seek medical help.^6^ This reticence exacerbates health disparities and challenges faced by men, underscoring the need for targeted public health strategies to address these critical issues.

Genitourinary health is a primary health concern in men. Despite its significance, there remains a reticence to openly address this issue because of persistent taboos. This is compounded by the prevalent attitude among men who often perceive themselves as impervious and indestructible. Thus, they tend to dismiss or ignore conditions that could cause discomfort.^9^ As a result, men shy away from discussing these issues, significantly impacting their quality of life and placing additional strain on the healthcare system.

Understanding the prevalence of genitourinary health problems in the population is necessary to efficiently tackle this problem.

## OBJECTIVE

This study aimed to assess the prevalence of ED and LUTS in middle-aged men and correlate these with quality of life data and sociocultural characterization.

## METHODS

This study is an observational, cross-sectional, and analytical investigation carried out between July 2021 and August 2022, following the Strengthening the Reporting of Observational Studies in Epidemiology (STROBE) guidelines.^10^ The research was conducted in Bauru, São Paulo, a city situated in the central-western region of the state. Bauru has an estimated population of 380,000 residents and a Human Development Index of 0.801. To ensure the representativeness of Bauru’s diverse population, a specific urban area was selected based on data provided by the municipal health department. This selected region has a population of 20,266 as estimated by the Brazilian Institute of Geography and Statistics (IBGE).^11,12^

The sample comprised middle-aged men residing in the selected neighborhood who completed all the questionnaires. Individuals under 18 years of age, middle-aged but not residing in the designated area, or who did not complete the questionnaires were excluded from the study.

Sample size was computed using confidence intervals. The confidence level was set at 95% and the margin of error was 5%. The estimated proportion of the population was calculated based on the prevalence of LUTS (69.0%) and ED (39.5%).^6,13^ The minimum calculated sample size was 368 individuals.

The sampling was conducted in two stages. First, the territory was divided into two large areas based on the service territory of the local health center. Next, a street at the study site was chosen as the starting point (Street 1) for each large area, and, from there, interviews were conducted sequentially on both sides of the road. The interviewers started with the streets in the vertical direction and then followed the streets in the horizontal direction. Data collection ended when all streets in the area were covered.

To ensure consistency in data collection, the study utilized Google Forms^®^ to standardize the instruments used. Additionally, interviewers were trained and calibrated through a meeting before data collection, explaining the details of our study, how to approach the interviewees, and addressing any potential uncertainties that may arise during the interview. Using smartphones, data were collected between July 2021 and August 2022 in men’s homes. The interview began after approaching the homes, identifying the participant, and the participants agreeing to participate by signing the informed consent form.

Instruments used for data collection were internationally validated questionnaires. In this study, the transcripts were converted to digital format according to the original layout in the following sequence:

1. Instrument for the sociocultural characterization of the participants: A questionnaire was prepared by the authors themselves with the aim of characterizing the individuals through variables such as age, self-declared color (white, black, yellow, brown, and indigenous), religion (Catholic, Evangelical, other, none), and marital status (single, married, stable union, widowed, separated, other).
2. ‘World Health Organization Quality-of-Life Scale (WHOQOL-BREF)’: A 5-point Likert scale developed by the WHO, in which quality of life is assessed through two questions on perception and satisfaction, as well as 24 other questions divided into four domains (physical, psychological, social relations, and environment), which, in the end, give a score of 0-100 for each domain.^14,15^
3. ‘International Prostate Symptom Score (IPSS)’: A scale widely used in the assessment of LUTS and voiding dysfunction and validated for the Portuguese language.^16^ The definition used to confirm the presence of LUTS was a score between 2 and 5 for any IPSS question (symptoms occurring less than half the time or more; or nocturia ≥ 2).^6,17^
4. ‘International Index of Erectile Function-6 (IIEF-6)’: A validated score for the assessment of sexual function that aids in the diagnosis and classification of ED.^18^ It has shown to be as effective as the IIEF-5 and IIEF-15 variants of the score.^19,20^

After data collection, the data was transported and analyzed using Google Spreadsheets^®^ and Jamovi^®^ software (Jamovi OpenStats, Sidney, Australia). Descriptive statistics were performed by age group (≤ 39 years; 40-59 years; ≥ 60 years). Spearman and Kruskal-Wallis correlation tests were used to analyze the sample and associations between the variables, given their assumptions. The significance level adopted for statistical analysis was 5%. In addition, the data are presented as median and interquartile range (IQR) based on the distribution pattern of the results.

This study was approved by the Research Ethics Committee (CEP), under protocol number 44434820.2.0000.5441, on June 30, 2023. The participants’ consent was obtained by signing the informed consent form.

## RESULTS

**Figure 1:**
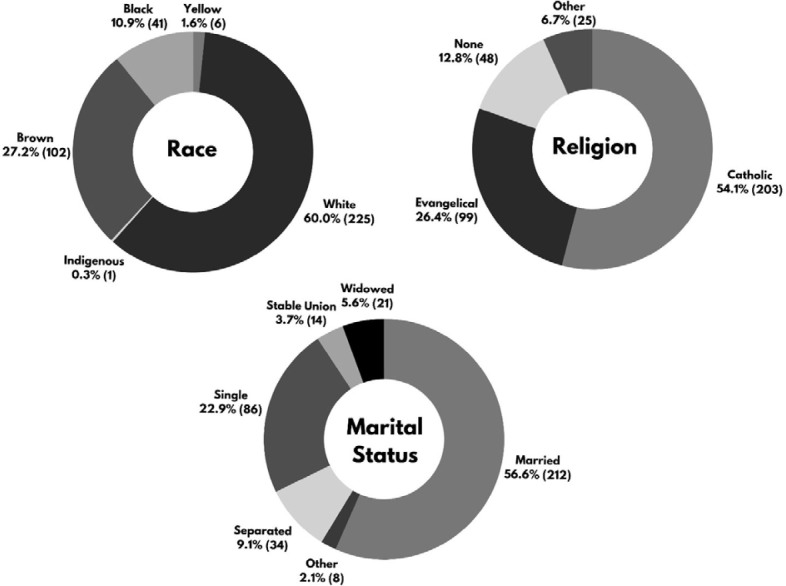
Sample characterization data.

A total of 375 men with a median age of 53.0 years (IQR = 38.5 – 66.0) participated in this study. Of those interviewed, 225 (60.0%) were white, 203 (54.1%) were Catholic, and 212 (56.6%) were married. Demographic data are shown in **Figure 1**.

In the IPSS questionnaire,^16^ the median score was 5 (IQR = 2–11), and the presence of LUTS, according to the criteria used in the study, was 257 (68.5%). In the IIEF-6 questionnaire,^18^ the median was 25.0 (IQR = 21–29) and 188 (50.1%) patients had some degree of ED. Data on the IPSS and IIEF-6 scores are presented in **Table 1**.

**Table 1:** Results of urological questionnaires divided by age

Questionnaire		≤ 39 years	40-59 years	≥ 60 years	Total
**IPSS**	Asymptomatic or mildly symptomatic (0-7)	84 (84.0%)	91 (70.0%)	68 (46.9%)	243 (64.8%)
	Moderately symptomatic (8-19)	14 (14.0%)	28 (21.5%)	56 (38.6%)	98 (26.1%)
	Severely symptomatic (20-35)	2 (2.0%)	11 (8.5%)	21 (14.5%)	34 (9.1%)
**IIEF-6**	Normal (26-30)	68 (68.0%)	81 (62.3%)	38 (26.2%)	187 (49.9%)
	Light (22-25)	19 (19.0%)	28 (21.5%)	36 (24.8%)	83 (22.1%)
	Mild to moderate (17-21)	10 (10.0%)	14 (10.8%)	29 (20.0%)	53 (14.1%)
	Moderate (11-16)	1 (1.0%)	7 (5.4%)	13 (9.0%)	21 (5.6%)
	Severe (1-10)	2 (2.0%)	0 (0.0%)	29 (20.0%)	31 (8.3%)

IPSS = International Prostate Symptom Score; IIEF-6 = International Index of Erectile Function-6.

Based on the first two questions of the WHOQOL-BREF questionnaire,^14^ health perception and satisfaction were assessed. Regarding perception, 279 (74.4%) participants considered it GOOD or VERY GOOD, and regarding satisfaction, 267 (71.2%) reported being SATISFIED or VERY SATISFIED (**Table 2**).

**Table 2:** Health perception and satisfaction measured by the World Health Organization Quality-of-Life Scale questionnaire

Question	Answers	≤ 39 years	40-59 years	≥ 60 years	Total
**1 - How would you rate your quality of life?**	Very bad	0 (0.0%)	1 (0.8%)	1 (0.7%)	2 (0.5%)
	Bad	4 (4.0%)	1 (0.8%)	5 (3.5%)	10 (2.7%)
	Neither bad nor good	18 (18.0%)	26 (20.0%)	40 (27.6%)	84 (22.4%)
	Good	48 (48.0%)	62 (47.7%)	63 (43.4%)	173 (46.1%)
	Very good	30 (30.0%)	40 (30.7%)	36 (24.8%)	106 (28.3%)
**2 - How satisfied are you with your health?**	Very dissatisfied	0 (0.0%)	3 (2.3%)	4 (2.8%)	7 (1.8%)
	Dissatisfied	4 (4.0%)	5 (3.9%)	19 (13.1%)	28 (7.5%)
	Neither satisfied nor dissatisfied	22 (22.0%)	29 (22.3%)	22 (15.1%)	73 (19.5%)
	Satisfied	44 (44.0%)	54 (41.5%)	66 (45.6%)	164 (43.7%)
	Very satisfied	30 (30.0%)	39 (30.0%)	34 (23.4%)	103 (27.5%)

Regarding the WHOQOL-BREF questionnaire,^14^ **Table 3** shows the four domains (physical, psychological, social relationships, and environment) that were converted into a scale of zero to 100 points according to the syntax recommended by the score.

**Table 3:** Median and quartiles of the quality of life domains measured by the World Health Organization Quality-of-Life Scale questionnaire

Domain	≤ 39 years	40-59 years	≥ 60 years	Total
Physical	83.3 (75.0-91.7)	79.2 (66.7-91.7)	70.8 (58.3-83.3)	75.0 (62.5-87.5)
Psychological	79.2 (70.8-87.5)	83.3 (75.0-91.7)	79.2 (66.7-83.3)	79.2 (70.8-87.5)
Social Relations	79.2 (66.7-93.8)	83.3 (75.0-97.9)	75.0 (58.3-83.3)	75.0 (66.7-91.7)
Environment	70.3 (62.5-81.3)	71.9 (59.4-84.4)	68.8 (59.4-78.1)	71.9 (59.4-81.3)

IPSS questionnaires^16^ and IIEF-6^18^ showed an inversely proportional correlation between their scores (P < 0.001). Furthermore, the presence and severity of the disorders measured by both questionnaires were inversely proportional to the final score of the quality of life domains measured by the WHOQOL-BREF.^14^ Finally, age had a significant correlation, directly proportional to the presence and severity of symptoms in the IIEF-6 and IPSS questionnaires.^16,17,18^ All data related to the urological questionnaires are shown in **Table 4**.

**Table 4:** Urological questionnaires correlated with quality of life and characterization data

	WHOQOL-BREF	Age	Other characterization data
IPSS	Physical (sp = -0.355; P < 0.001) Psychological (sp = -0.237; P < 0.001) Social relations (sp = -0.242; P < 0.001) Environment (sp = -0.230; P < 0.001)	sp = 0.383 P < 0.001	Color (Kw = 2.64; P = 0.619) Religion (Kw = 3.95; P = 0.267) Marital status (Kw = 25.5; P < 0.001)
IIEF-6	Physical (sp = 0.390; P < 0.001) Psychological (sp = 0.269; P < 0.001) Social relations (sp = 0.363; P < 0.001) Environment (sp = 0.248; P < 0.001)	sp = -0.460 P < 0.001	Color (Kw = 5.98; P = 0.201) Religion (Kw = 8.03; P = 0.045) Marital status (Kw = 23.4; P < 0.001)

WHOQOL-BREF = World Health Organization Quality-of-Life Scale; sp = Spearman’s test; Kw = Kruskal–Wallis test; IPSS = International Prostate Symptom Score; IIEF-6 = International Index of Erectile Function-6.

Among the characterization data, only marital status showed a significant correlation with the data collection instruments, as shown in **Table 5**.

**Table 5:** Data from urological questionnaires and median age divided by marital status

	Age Median (IQR)	IPSS Median (IQR)	IIEF-6 Median (IQR)
Single	30.5 (23.2-42.8)	3.0 (1.0-6.0)	27.0 (23.0-29.0)
Married	55.5 (44.0-68.0)	4.5 (1.0-11.0)	25.0 (21.0-29.0)
Other	48.5 (33.2-62.2)	7.5 (3.8-11.8)	23.0 (20.8-28.2)
Widowed	74.0 (69.0-77.0)	10.0 (6.0-15.0)	17.0 (10.0-24.0)
Separate	60.5 (53.8-66.0)	8.0 (4.2-18.0)	23.0 (18.2-28.0)
Stable Union	46.5 (34.2-61.8)	8.5 (4.5-18.0)	27.0 (24.5-28.8)

IPSS = International Prostate Symptom Score; IIEF-6 = International Index of Erectile Function-6; IQR = interquartile range.

## DISCUSSION

Men’s health, constrained by the scope of promoting and preventing health issues, requires targeted attention. Nonetheless, men frequently seek professional healthcare only upon encountering health problems, often due to a confluence of factors, including sex and cultural, personal, physical, and historical influences.^21,22,23^

This cross-sectional study, conducted in a municipality in the countryside of the state of São Paulo, is the first population-based epidemiological study on LUTS and ED in middle-aged men related to the quality of life in the region. By implementing epidemiological surveys that utilize efficacious instruments, researchers can elucidate the dynamics of diseases and health. When integrated with local political and social frameworks, these insights contribute to the formulation of public policies with the potential to significantly alter health practices at the population level.^24,25^

In the present study, it was noted that the demographic profile of the men surveyed reflects sociocultural characteristics that align with the contemporary historical context of Brazil’s southeast region: predominantly white, Catholic, and married (**Figure 1**).^11,26,27,28,29^ This finding is essential for analyzing the extent of disparity across diverse ethnic, social, and cultural groups, considering that these disparities are frequently rooted in historical determinants with direct repercussions on overall health outcomes.^30^

The survey findings indicated a significant prevalence of LUTS and ED (**Table 1**). Moreover, these conditions were directly associated with quality of life assessments, as gauged by the WHOQOL-BREF instrument.^14^ Additionally, a correlation existed between these disorders and various demographic factors, including marital status and age.

Regarding LUTS, studies have estimated a 45.0% to 62.5% prevalence in middle-aged men over 18 years old, adhering to the International Continence Society criteria.^6,17^ However, this study revealed a greater prevalence of 68.5% in middle-aged men, signifying a higher incidence of LUTS in the surveyed group compared to other national and international data, highlighting its diverse impact in Brazil.

The observed prevalence of ED in this sample was 50.1%, aligning closely with national research findings, such as 45.9% in a Santos study and 46.2% across various Brazilian regions.^31,32^ These figures are also consistent with international research, such as the Massachusetts Male Aging Study that identified a 52% prevalence; a survey in Boston, Massachusetts that noted 40%-70% prevalence;^7^ a French study that reported 39% prevalence among men aged 18-70 years;^33^ and recent UK research that observed a 41.5% prevalence in men aged > 18 years.^34^ Additionally, this study corroborates earlier research indicating an age-related increase in ED prevalence.

There is an evident analytical deficiency in examining the intersection of quality of life with urological assessments. Limited research correlating conditions such as LUTS and ED demonstrates congruence with the present study, affirming the profound effect on individual quality of life.^35,36.37^ Quality of life in this study was interpreted through individual self-assessment of life circumstances, cultural contexts, and value systems against their aspirations and concerns. From the perspective of healthcare professionals, quality of life may signify enhanced health outcomes within individual, societal, and cultural frameworks.^14,38^ This align with the intricate nature of men’s perceptions of their health, masculinity, and general well-being. The highlighted prevalence of these disorders in this study, coupled with their considerable impact on the quality of life, underscores the urgency for further investigation in this field and heightens the necessity for health services at all levels to prioritize these health issues, recognizing their potential widespread influence on individuals’ lives.

Finally, previous studies have rarely explored the relationship between marital status and urological health. Nonetheless, this research discovered that, particularly among widowers and separated men, there was a higher incidence and more severe symptoms reported in the IIEF-6 and IPSS assessments.^16,17,18^ This evidence supports the theory that the presence of a partner may increase the likelihood of men seeking medical attention, thus potentially improving their self-care practices.^39^ These insights should be approached with prudence and further investigated, as the older age of widowed and separated male participants could represent a confounding factor in this analysis due to the presence of age-related senility, which can contribute to higher rates of ED and LUTS. Therefore, it has not been conclusively determined whether advancing age or marital status is the primary factor influencing the prevalence of genitourinary issues.

### Merits and limitations

Elucidating the health profile of men is imperative for the development of public health policies and informed distribution of resources based on the collected data. This study provides novel insights into the prevalence of specific health conditions among middle-aged men, predominantly those aged 18 to 39 years, a demographic often underrepresented in existing research despite a growing incidence of such concerns among younger individuals.^17,40,41,42^ It has been determined that the presence of ED and LUTS in men aged ≥ 18 years is intricately associated with the deterioration of the participants’ quality of life, given the substantial prevalence and direct correlation to various facets of an individual’s well-being. This study is unprecedented in examining the prevalence of these conditions within the local context of a municipality. These findings underscore the necessity for an integrated approach to health interventions, recognizing that these issues substantially influence a broader spectrum of men’s health.

Concerning the limitations of this study, there are issues related to the generalizability of the results. The main limitation is the inability to confirm causal relationships inherent in cross-sectional studies, followed by the inability to perform a multivariate analysis to assess the extent to which the quality of life is affected by LUTS or ED, considering the simultaneous effect of age. Our sample contained several cells with zero or very few observations (< 5), which could have significantly reduced the statistical power of the multivariate tests. Another limitation was the use of self-reported measures for ED and LUTS. This study did not include the levels of education and occupation; therefore, these may be relevant variables for future research. Finally, it was not feasible to include other regions of the municipality during the data collection period because the COVID-19 pandemic was advancing during the data collection period. Future studies with larger sample sizes are required to explore the potential correlation between age and quality of life in relation to LUTS and ED.

## CONCLUSION

The prevalence of ED and LUTS in middle-aged men is high. Additionally, age and marital status were significantly correlated with these disorders. Finally, the presence or severity of these disorders is inversely correlated with the individual’s quality of life.

This high prevalence highlights the urgent need for targeted public health interventions. Health professionals should consider these findings when developing health promotion and disease prevention strategies such as educational campaigns to reduce the stigma associated with discussing genitourinary health in men. This could encourage men to seek timely medical advice and guide them toward a better quality of life.
